# Acute stroke in the emergency department: A chart review at KwaZulu-Natal hospital

**DOI:** 10.4102/safp.v62i1.5126

**Published:** 2020-08-20

**Authors:** Steve G. Feris, Bavani Naicker

**Affiliations:** 1 Division of Emergency Medicine, School of Clinical Medicine, University of KwaZulu-Natal, Durban, South Africa

**Keywords:** stroke, emergency medicine, rural health, primary care, neurology

## Abstract

**Background:**

Stroke is the second leading cause of death worldwide. There is limited literature detailing the clinical profile of stroke in developing countries’ emergency departments (EDs). The aim of this study is to describe the demographics and risk factors of patients presenting with stroke to an ED in South Africa.

**Methods:**

This study was a retrospective chart review of all patients with a clinical diagnosis of stroke presenting to an ED of a regional hospital in rural KwaZulu-Natal from November 2018 to November 2019.

**Results:**

A total of 362 patient charts were screened, and 136 of the charts met the inclusion criteria for the study. Seventy per cent of the patients had pre-existing hypertension, only one patient was not on treatment and two patients were not on secondary prevention. In human immunodeficiency virus–positive patients (20.5%; *n* = 28), 17 patients were under 50 years old. The most common finding on computer tomography was ischemic strokes (74%, *n* = 100). Thrombolysis was given to five patients included in the study. The overall in-hospital mortality rate was 4.06%.

**Conclusion:**

This study adds to the limited data about stroke in South Africa. Our population represents a unique blend of infectious and lifestyle disease. More research in this setting is recommended to develop local guidelines on emergency stroke care.

## Introduction

Stroke is the second leading cause of death globally, after coronary artery disease, with a total of 6.3 million deaths in 2015. Approximately half of stroke sufferers die within 1 year, the majority of whom are over 65 years old.^1^ In South Africa, stroke is the third leading cause of death.^2^

The stroke burden is disproportionately greater in rural South Africa.^3^ Despite comprising only 20% of the population, rural South Africa carries at least half of the stroke burden.^3^ The Southern African Stroke Prevention Initiative study showed that the prevalence of stroke in rural South Africa is estimated to be 300 per 100 000.^4^

Risk factors for stroke include age, hypertension, human immunodeficiency virus (HIV), dyslipidaemia, diabetes mellitus, smoking, obesity, previous cerebral vascular disease and cardiac diseases.^5^ South Africa has an 18% prevalence of HIV, which complicates stroke management.^6^

Stroke management starts at the primary health care level with prevention for patients at risk. Most South Africans collect their medications from primary care clinics. Once a patient has had a stroke, dedicated stroke units have been shown to improve outcomes.^7^ Intravenous thrombolytic reperfusion therapy is available in South Africa if a patient presents within 4.5 hours from symptom onset.^7^

Rehabilitation therapy has been shown to produce outcomes equal to that of reperfusion at 6 months post stroke. South Africa has a limited number of allied health professionals to provide rehabilitation services, making reperfusion a more attractive option in the South African context. Endovascular techniques have been performed in South Africa, but this is limited to neuro-interventional centres and a highly specific patient population.^7^

By 2030, there will be an estimated 20 million deaths per year because of stroke. Developing countries have limited epidemiological data yet 80% of global stroke burden occurs in these settings.^8^ Developed countries spend a total of 3% of the health budget on stroke care. The exact cost of stroke care in South Africa is not known but one study estimated a total of R2.5 – R4.2 million in 2012 or 1.6% – 3% of the sub-district health expenditure, majority of which was incurred by inpatient costs.^9^

Improving stroke care in South Africa will require rigid primary prevention programmes, streamlined access to stroke ready units and more rehabilitation centres. To begin tackling each of these aspects, more local data are needed to plan and budget appropriately.

This study aims to describe the demographics and risk factors of patients presenting with stroke to an emergency department (ED) in South Africa.

## Methods

This study was a retrospective chart review of all patients with a clinical diagnosis of stroke presenting to an ED of a regional hospital in rural KwaZulu-Natal from November 2018 to November 2019.

The ED provides regional-level service to a population of approximately 600 000 in the iLembe district and supports three district hospitals. It is a stroke ready centre, staffed with specialist emergency and family physicians, with access to computer tomography (CT) and onsite specialist physicians. There is no neurology and neurosurgery service.

All patient charts with a clinical diagnosis of stroke or a neurological complaint were screened for inclusion. The Cincinnati Pre-hospital Stroke Scale (CPSS) in combination with clinician assessment reflected in the chart was used as screening tools for inclusion.^10^

Patients under the age of 12 years with stroke were managed in a separate paediatric centre and were therefore not included.

Clinical details and demographical information were entered onto a spreadsheet. Additional information was collected from the Radiology Department reports and from the Internal Medicine Department discharge summaries. If the required data were still incomplete, then the patient was excluded from the study.

The charts were analysed for patient demographics, co-morbidities, chronic medications, triage vital signs, CT findings and discharge plan of patient from the ED.

Data were collected using Microsoft Office Excel and analysed using Statistical Product and Service Solutions (SPSS). Descriptive measures were used to describe the clinical and demographical findings.

### Ethical consideration

Ethical approval was given by the University of KwaZulu-Natal Biostatistics Research Ethics Committee (BREC reference: BREC/000000832/2019) and the KwaZulu-Natal Department of Health (KZ_202001_013).

## Results

**FIGURE 1:**
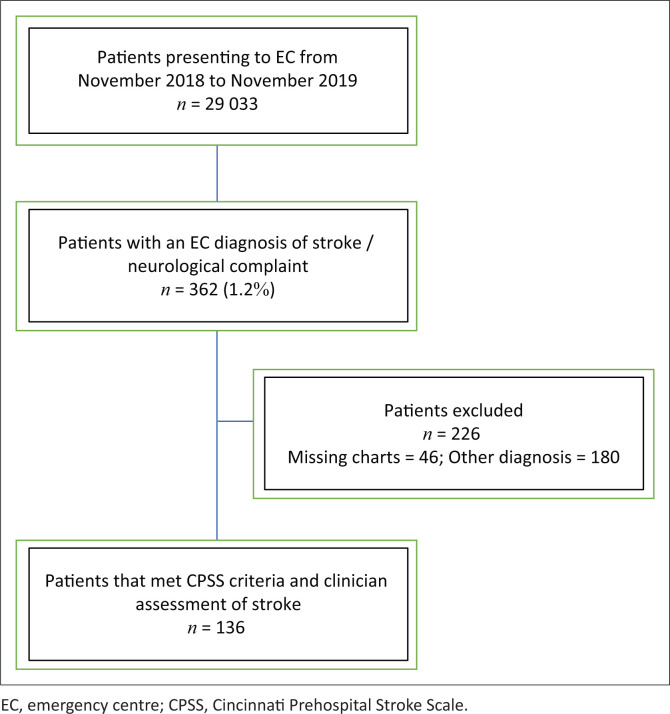
Breakdown of study sampling.

Figure 1 provides a breakdown of chart screening and inclusion. A total of 29 033 patients visited the ED during the study period. Three-hundred sixty-two patients presented with neurological complaints, and they were screened for inclusion in the study. Two-hundred twenty-six patients were excluded, and 180 patients did not meet CPSS criteria and had a diagnosis other than stroke. The remaining 46 patients were excluded for missing charts and insufficient data in their discharge and radiology reports. The final study sample included 136 patients.

Table 1 shows demographical data and risk factors of patients presenting with stroke. The average age was 59.8 years with standard deviation ± 15.3 years. Twenty-nine (21.32%) patients were aged below 50 years. Of those younger than 50 years, 17 (58.62%) patients were HIV positive, compared to the HIV prevalence of 20.58% in the total sample.

**TABLE 1:** Sample demographic data.

Variable	*n*	% of patients
Male	52	38.24
Female	84	61.76
0–20 years	2	1.47
21–40 years	13	9.55
41–60 years	46	33.82
61–80 years	65	47.79
81–100 years	9	6.61
> 100 years	1	0.74
Hypertension	100	73.52
Diabetes mellitus	58	42.65
Atrial fibrillation	4	2.94
Previous stroke	44	32.35
Ischemic heart disease	4	2.94
HIV	28	20.58
Dyslipidaemia	35	25.73
Smoking	5	3.67

*n* = 136.

HIV, human immunodeficiency virus.

Table 2 is a summary of the triage office’s clinical assessment on presentation to the ED. Most patients were triaged as yellow codes (*n* = 110, 80.9%), followed by orange (*n* = 23, 16.9%) and red (*n* = 3, 2.2%) codes.

**TABLE 2:** Clinical presentation and triage scores.

Variable	Mean	Standard deviation	Sample range	Frequency	
				*n*	%
Systolic blood pressure (mmHg)	146.53	33.64	91–269	-	-
Pulse (beats/min)	86.11	15.68	47–129	-	-
Respiratory rate (breaths/min)	18.80	3.50	12–32	-	-
Oxygen saturation (SpO^2^%)	96.63	2.08	84–100	-	-
Blood glucose (mmol/l)	8.08	4.14	3.2–30	-	-
Temperature (°C)	36.11	0.34	35–37	-	-
**South Africa Triage Scale Colour**					
Red	-	-	-	3	2.21
Orange	-	-	-	23	16.91
Yellow	-	-	-	110	80.88
Green	-	-	-	0	0.00
**Alert Verbal Pain Unresponsive Scale**					
Alert	-	-	-	133	97.79
Verbal	-	-	-	0	0.00
Pain	-	-	-	2	1.47
Unresponsive	-	-	-	1	0.74

The common CT findings identified were infarctions (*n* = 100, 73.5%) followed by haemorrhages (*n* = 4, 2.9%). Only two CTs showed tumours and one revealed hydrocephalus. Thrombolysis was given to five patients included in the study and seven others during the study period who were excluded for missing charts.

Table 3 shows how many patients were on treatment for their co-morbidities. The disposition pathway was usually to internal medicine (*n* = 123, 90.4%). Only eight (5.8%) patients were transferred to a central hospital’s neurology unit. The mortality rate of patients admitted to the study hospital was 5/123 (4.06%). No deaths were reported among the patients transferred out; however, charts from the neurology unit were not reviewed to confirm outcome. Access to these charts falls outside the ethical permissions granted for this study.

**TABLE 3:** Co-morbidities and treatment.

Co-morbidity	Diagnosed		On treatment	
	*n*	%	*n*	%
Hypertension	100	73.53	99	72.7
Diabetes Mellitus	58	42.65	56	41.1
Atrial Fibrillation	4	2.94	2	1.4
Ischemic Heart Disease	4	2.94	4	2.9
HIV	28	20.59	28	20.5
Dyslipidaemia	35	25.74	98	72.0
Atherosclerotic CVD risk†	n/a	-	98	70.5

HIV, human immunodeficiency virus; CVD, cardiovascular disease; n/a, not applicable.

† , Patients on preventative medication because of their atherosclerotic cardiovascular disease risk.

## Discussion

During the study period, 29 033 patients visited the ED; 136 (0.47%) of these patients had stroke or neurological complaints that met study criteria. This is comparable to a similar study conducted at a tertiary hospital ED in Johannesburg which saw approximately 60 000 patients per annum and included 0.39% of their patients.^11^

Most of the patients (*n* = 107, 78.68%) presenting with stroke were above 50 years old. This is similar to international trends. This contrasts with a local study in a rural setting where stroke is most prevalent in the 45–59 years age group.^3^

In this study, 28 (20.58%) patients were HIV positive, and 17 (12.5%) patients were under 50 years old. A study conducted in Cape Town showed a 6.1% HIV rate in stroke patients, of which 91% of patients were under 46 years old.^12^ There is a growing trend of younger patients presenting with HIV and stroke in South Africa.

Adding to the infectious disease burden, the prevalence of lifestyle-related diseases is on the rise in the younger South African population. These are well-known significant risk factors for the development of stroke.^13^ In our patients aged below 50 years with stroke, 10 (20%) patients were hypertensive, 6 (12%) patients were diabetic and 7 (14%) patients had previous stroke.

Our population has a unique blend of HIV and non-communicable disease putting them at risk for stroke at a much younger age. Of our 17 HIV-positive patients under the age of 50 years, three patients were hypertensive, two patients had previous strokes and one patients was diabetic.

In all patients with known risk factors, most were on treatment (Table 3); however, no measure of control or adherence was included in the study such as serum markers and previous recordings. It was noted that 63 out of 98 patients were on a statin despite not having a diagnosis of dyslipidaemia, likely as a preventative measure for atherosclerotic cardiovascular events.

Intravenous thrombolytics were given to only five (3.68%) patients in this study. This is likely to time delays^11^ and strict inclusion criteria for thrombolysis.^14^

Globally, ischemic events are the most common cause of stroke and hypertension, the most common risk factor.^15^ This was the same in our patient cohort as well. Atrial fibrillation is known to increase stroke risk fivefold;^16^ however, in this study, only four patients had atrial fibrillation.

In a recent study^17^ of South African hospitals’ stroke data, hypertension was present in only 49.8% of the confirmed stroke patients versus 73.52% in our study.

Eight patients were treated in a dedicated stroke unit at a neurology centre.

This study was of a small retrospective sample and encompasses all the inherent flaws with this study design. There is total reliance on the accuracy of the data recorded, and not being able to retrieve all the requested charts may influence findings. The quality of notes, rank and experience of the attending clinician may also influence retrospective findings.

## Conclusion

This study adds to the current trend of African data showing stroke in younger patients, with both HIV and non-communicable disease. It also reaffirms the belief that primary prevention and risk factor control are the biggest weapons in the fight against stroke. Although only five patients were given thrombolysis, this is significant in that it happened at a rural hospital and gives an encouraging sign of the potential of regional-level hospitals in rural South Africa.

National guidelines recommend acute strokes be managed in an established network involving all levels of care including a prehospital directive to avoid all delays in accessing stroke care.^18^ The iLembe district needs more clearly refined local pathways to implement this recommendation and the logistics involved. Stroke places a significant load on the global health system, social support structures and economy. More research is required to quantify the impact of stroke across all sectors in South Africa.
